# Higher incidence of penetrating keratoplasty having effects on repeated keratoplasty in South Korea: A nationwide population-based study

**DOI:** 10.1371/journal.pone.0235233

**Published:** 2020-07-06

**Authors:** Kyoung Yoon Shin, Dong Hui Lim, Kyungdo Han, Tae-Young Chung

**Affiliations:** 1 Department of Ophthalmology, Samsung Medical Center, Sungkyunkwan University School of Medicine, Seoul, Republic of Korea; 2 Department of Clinical Research Design & Evaluation, Samsung Advanced Institute for Health Sciences and Technology, Sungkyunkwan University, Seoul, Republic of Korea; 3 Department of Statistics and Actuarial Science, Soongsil University, Seoul, Republic of Korea; Universidad de Monterrey Division de Ciencias de la Salud, MEXICO

## Abstract

**Purpose:**

To investigate the incidence of corneal transplantation and identify rates and risk factors of repeated corneal transplantation in South Korea.

**Methods:**

This is a retrospective population-based cohort study using the Korean National Health Insurance System database. Among the entire South Korean population (N = 51,827,813), we included those who underwent corneal transplantation more than once between January 2006 and December 2016, and analyzed the annual incidence of keratoplasty. The person-year incidence of repeated keratoplasty after the first operation was calculated according to risk factors including age group, sex, income level, surgical method, surgical etiology, and presence of major systemic diseases. Cox regression analysis was employed to evaluate the hazard ratios of those risk factors on repeated keratoplasty.

**Results:**

A total of 9,452 cases of corneal transplantation occurred from January 2006 to December 2016. The average annual incidence of corneal transplantations was 1.694 per 100,000. The proportion of penetrating keratoplasty steadily decreased from 92.22% in 2006 to 77.81% in 2016. The average incidence of repeated keratoplasty among those who underwent corneal transplantation at least once was 43.24 per 1,000 person-years. Males had a greater incidence of repeated keratoplasty compared to females (males: 47.66 per 1,000, females: 36.04 per 1,000). The age group from 20 to 39 years demonstrated the lowest incidence of repeated keratoplasty at 24.94 per 1,000. Keratoconus had the lowest incidence of repeated keratoplasty (22.82 per 1,000).

**Conclusion:**

This study may provide a better understanding of corneal diseases, help predict disease burden, and plan health care systems accordingly in South Korea.

## Introduction

The spectrum of causative corneal diseases differs by country and ethnicity, and the prevalence of corneal blindness is diverse in each geographical area [1]. For instance, Fuch’s dystrophy is often encountered in the Unites States and Western countries, while keratoconus is much more prevalent in the Middle East, and infectious keratitis is the most common reason for keratoplasty in developing countries in Southeast Asia [2–4]. Accordingly, the overall rate of corneal transplantation and the proportion of implemented surgical techniques (penetrating keratoplasty, anterior lamellar keratoplasty, and endothelial keratoplasty) vary from country to country since they are affected by factors such as prevalence and causes of corneal blindness, as well as by socioeconomic parameters such as accessibility to health care and corneal procurement rate [5]. The rate of corneal transplantation is significantly higher in the United States compared to other countries, and Western countries have a higher proportion of endothelial keratoplasty, since Fuch’s dystrophy is much more prevalent compared to Eastern countries [2]. Differences in causative corneal diseases and implemented surgical techniques influence the outcome of corneal transplantation and affect the rate of repeated corneal transplantation. Thus, the rate and incidence of repeated corneal transplantation also vary by country and ethnicity.

Although there have been many studies on causes of corneal blindness and incidence of corneal transplantation and repeated corneal transplantation in developed Western countries, relatively few have evaluated trends of corneal transplantation in Asian populations.

In the current study, we investigated the incidence of corneal transplantation and repeated corneal transplantation in South Korea from 2006 to 2016, and identified specific risk factors for repeated corneal transplantation using data from a nationwide health care registry.

## Methods

### Study setting

This was a nationwide, population-based study using data from the Korean national health claims database of patients who underwent keratoplasty. We accessed health claims records with the National Health Insurance Service (NHIS) of South Korea from 2006 through 2016. The Korean NHIS is a mandatory public health insurance system that provides universal health coverage to all Koreans except Medicaid beneficiaries in the lowest income bracket, around 3% of the population. The NHIS database includes all health care utilization information for inpatient and outpatient visits including diagnoses, dates of visits, procedures, prescription records, comorbidities, and demographic information of the entire South Korean population. Patients can be identified easily by a unique Korean Resident Registration Number; this guarantees absence of duplication or omission of data. This database has been used in numerous epidemiologic studies, and the details have been previously described [6,7]. This study was performed in accordance with the principles of the Declaration of Helsinki and approved by the Samsung Medical Center Institutional Review Board (SMC 2018-03-144).

### Incidence of corneal transplantation

Patients who underwent penetrating keratoplasty (registration code S5372) and lamellar keratoplasty (S5371 and S5374) during the eleven-year study period (from 2006 to 2016) were included in incidence estimates. All surgical codes were registered by surgeons. The incidence rate (IR) in each specific year was estimated as the number of keratoplasty cases that year divided by the total population of the year registered in the NHIS database. The IR of corneal transplantation was estimated according to surgical method, age group, sex, and etiology for corneal transplantation.

### Identification of repeated corneal transplantation

We identified repeated case of keratoplasty as follows. Patients who underwent keratoplasty more than once during the study period were defined to have underwent repeated keratoplasty. The first surgery was regarded as initial keratoplasty and later surgeries were regard as repeated keratoplasty cases. Patients having diagnostic code of corneal transplant status (registration code Z94.7) before the first corneal transplantation between 2006 and 2016 were regarded to have undergone keratoplasty before 2006 and the surgeries of them were counted as repeated keratoplasty.

### Risk factors for repeated corneal transplantation

Patients who underwent keratoplasty before 2006 (having Z94.7 code before the first surgery during the study period) were excluded from the analysis identifying risk factors for repeated keratoplasty. Baseline characteristics of age at first keratoplasty, sex, patient income level, initial surgical method, initial surgical indication, and other systemic diseases were compared between patients who underwent keratoplasty once and those who underwent repeated keratoplasty. The person-year IR of repeated keratoplasty after the first operation was calculated according to age group, sex, surgical method, initial surgical indication, and presence of other systemic diseases. To evaluate the hazard ratio (HR) of repeated keratoplasty and its 95% confidence interval (CI), we employed Cox regression analysis. Event was defined as repeated keratoplasty, and the time period was defined as the time between first keratoplasty and repeated keratoplasty or that between first keratoplasty and last follow-up date. HR of repeated keratoplasty by risk factors stated above. Subjects with income less than the lower 20 percentile were defined to have low income.

Statistical Analysis System software version 9.4 (SAS Inc. Cary. NC) was used for all analyses.

## Results

### Incidence of corneal transplantation

**Fig 1:**
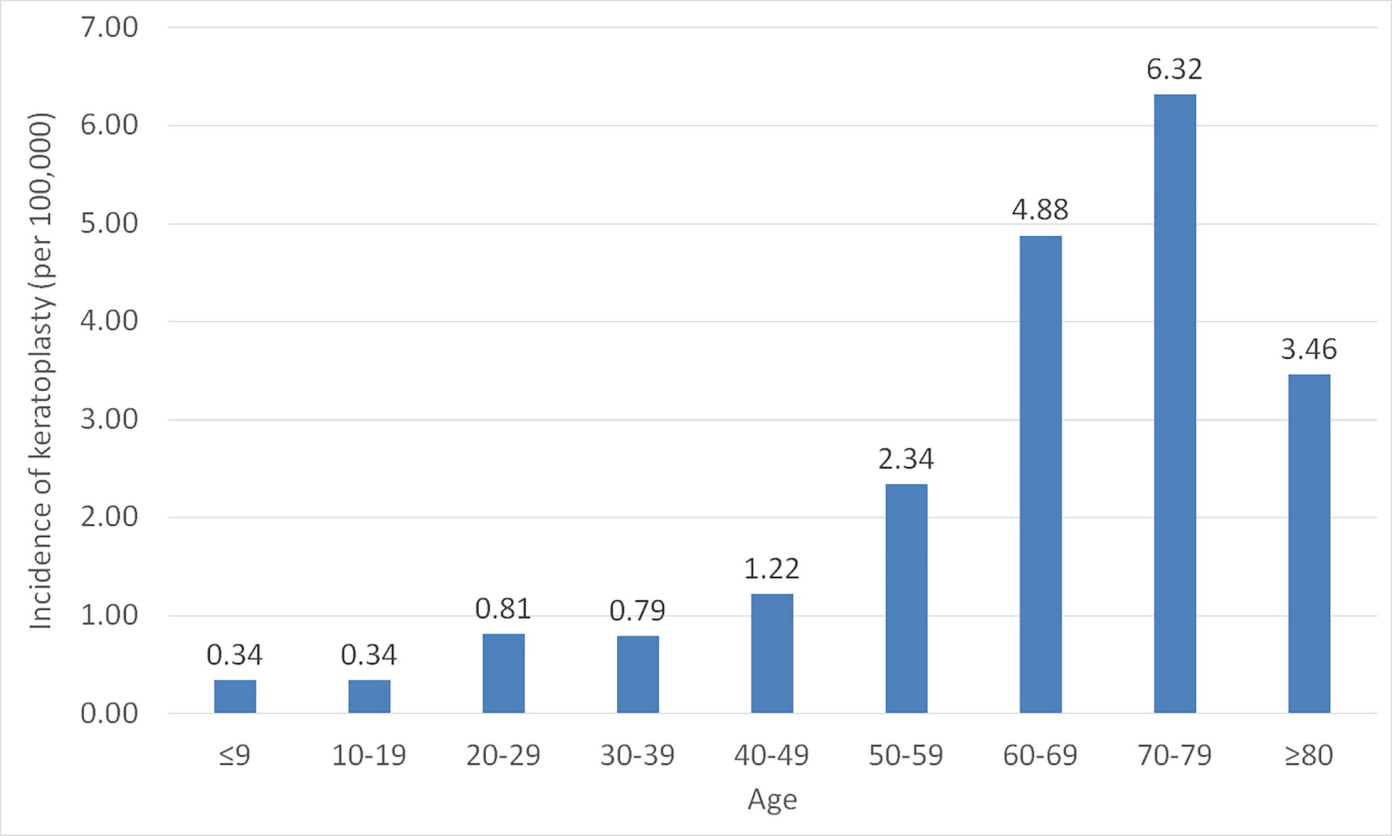
Average incidence of keratoplasty from 2006 to 2016 by age group. The age group of 70–79 years demonstrated the highest annual incidence of keratoplasty (6.32 per 100,000 people).

The number of overall cases of keratoplasty in the general population from 2006 to 2016 was 9,452. Table 1 demonstrates annual trends of keratoplasty incidence; the annual incidence ranged from 1.4488 to 1.9516 cases per 100,000 people throughout the study period. Penetrating keratoplasty accounted for the majority of keratoplasty cases. The annual incidence of lamellar keratoplasty increased from 0.1235 cases per 100,000 people in 2006 to 0.4089 cases per 100,000 people in 2016. In 2016, penetrating keratoplasty accounted for 77.81% (754 cases) and lamellar keratoplasty accounted for 22.19% (215 cases) of all keratoplasty cases. The peak annual incidence age shifted from 60~69 in 2006 (5.3683 cases per 100,000 people) to 70~79 in 2016 (7.2779 cases per 100,000 people). The average incidence of keratoplasty from 2006 to 2016 grouped by age is listed on Fig 1 and peaked at 70–79 years of age. Males had a greater incidence of keratoplasty compared to females. The number of keratoplasty according to surgical indication is also listed in Table 1. Bullous keratopathy was the leading cause of corneal transplantation.

**Table 1:** Case incidence of corneal transplantation in South Korea from 2006 to 2016 (annual incidence per 100,000 people).

	2006	2007	2008	2009	2010	2011	2012	2013	2014	2015	2016
N	48371311	48603519	49560378	49884458	50166793	50445164	50763154	51013675	51281917	51574044	51827813
Event	771	718	796	979	847	943	888	812	861	868	969
Overall IR	1.5864	1.4488	1.5958	1.9516	1.6792	1.8578	1.7409	1.5836	1.6697	1.6750	1.8429
**Surgical method**											
• PKP	1.4629	1.2874	1.3993	1.7523	1.4909	1.6391	1.5546	1.3418	1.4214	1.3315	1.4340
• LKP	0.1235	0.1614	0.1965	0.1993	0.1883	0.2187	0.1862	0.2418	0.2482	0.3435	0.4089
**Age**											
-9	0.5565	0.2280	0.3928	0.3452	0.3574	0.2565	0.4709	0.3455	0.1954	0.4113	0.2165
10–19	0.4794	0.3817	0.4101	0.2641	0.3670	0.4499	0.2486	0.2733	0.3177	0.2802	0.2553
20–29	1.0579	0.9402	0.8961	1.0591	0.5437	1.1210	0.8408	0.5479	0.7183	0.5668	0.5855
30–39	0.8468	0.7864	0.7340	0.8473	0.9023	0.7923	0.9216	0.8622	0.7300	0.6519	0.6556
40–49	1.3463	1.1787	1.4341	1.4456	1.3336	1.4081	1.2153	0.9772	1.1977	0.8793	0.9918
50–59	2.2569	2.3690	2.5272	2.8259	2.4589	2.2755	2.2334	1.8890	1.9003	2.4005	2.5614
60–69	5.3683	4.6865	4.6539	6.7010	5.2257	5.2121	4.7696	4.6770	4.3707	3.7722	4.2904
70–79	5.2102	4.3968	5.5232	7.2163	6.1559	7.4879	6.3075	6.0642	6.8518	7.0744	7.2779
80–89	1.9997	3.0328	2.9466	3.7551	2.6575	3.9947	4.2704	3.5398	3.4963	3.7235	4.6907
**Sex**											
• Male	2.0373	1.8263	2.0628	2.5448	2.1637	2.3592	2.2478	2.0574	2.2007	2.0982	2.3201
• Female	1.1319	1.0689	1.1260	1.3552	1.1923	1.3543	1.2327	1.1088	1.1375	1.2512	1.3640
**Indication of Surgery**											
• Keratoconus	0.2490	0.2563	0.1844	0.1914	0.1626	0.1832	0.1882	0.1248	0.1745	0.1775	0.2358
• Dystrophy	0.0782	0.0404	0.0581	0.0658	0.0714	0.0926	0.0882	0.0936	0.0834	0.0830	0.1179
• Trauma	0.1914	0.1897	0.2165	0.3229	0.2399	0.2699	0.2803	0.2282	0.3006	0.3184	0.3537
• Congenital and tumor	0.0473	0.0323	0.0521	0.0478	0.0377	0.0335	0.0451	0.0566	0.0524	0.0656	0.0513
• Bullous Keratopathy	0.3457	0.3975	0.4551	0.5602	0.4818	0.5300	0.4862	0.4603	0.4305	0.4323	0.4812
• Infectious Keratitis	0.2675	0.2159	0.2085	0.2891	0.2498	0.2778	0.2019	0.2067	0.1939	0.1698	0.1445
• Corneal opacity	0.2078	0.1352	0.1504	0.1694	0.1487	0.1379	0.1176	0.0975	0.0911	0.0637	0.0609
• Others	0.0144	0.0081	0.0160	0.0179	0.0159	0.0099	0.0098	0.0117	0.0116	0.0077	0.0095
**Number of Surgery**											
• Initial KP	1.4012	1.2753	1.3412	1.6646	1.4076	1.5347	1.4174	1.2793	1.3380	1.3180	1.4549
• Repeated KP	0.1852	0.1735	0.2546	0.2871	0.2716	0.3231	0.3235	0.3042	0.3316	0.3570	0.3880

N = total population in South Korea; IR = incidence rate; PKP = penetrating keratoplasty; LKP = lamellar keratoplasty.

### Incidence and risk factors for repeated corneal transplantation

Table 2 compares baseline characteristics between patients who underwent keratoplasty only once and more than once. Among 5,180 patients, 1,308 underwent repeated corneal transplantation. The proportion of males was higher in the repeated keratoplasty group (61.31% in the non-repeated keratoplasty group vs. 68.27% in the repeated keratoplasty group, p<0.0001). The initial surgical method (whether penetrating or lamellar keratoplasty) did not show significant difference between two groups. Unlike other initial surgical indication, keratoconus presented much lower proportion in repeated keratoplasty group.

**Table 2:** Demographics of study subjects who underwent keratoplasty from 2006 to 2016 according to whether repeated keratoplasty.

		Repeated keratoplasty		
	Total	No	Yes	p-value*
**N**	5180	3872	1308	
**Age**	53.3 ± 18.7	52.9 ± 19.2	54.4 ± 17.1	0.0137
**Age group**				< .0001
-19	287 (5.54)	219 (5.66)	68 (5.2)	
20–39	892 (17.22)	741 (19.14)	151 (11.54)	
40–59	1688 (32.59)	1199 (30.97)	489 (37.39)	
60–79	2156 (41.62)	1580 (40.81)	576 (44.04)	
80-	157 (3.03)	133 (3.43)	24 (1.83)	
**Sex (male)**	3267 (63.07)	2374 (61.31)	893 (68.27)	< .0001
**Income (Low)**	1267 (24.46)	962 (24.85)	305 (23.32)	0.2667
**Initial Surgical Method**				0.1824
• Penetrating keratoplasty	4437 (85.66)	3302 (85.28)	1135 (86.77)	
• Lamellar keratoplasty	743 (14.34)	570 (14.72)	173 (13.23)	
**Indication**				< .0001
• Keratoconus	709 (13.69)	597 (15.42)	112 (8.56)	
• Dystrophy	256 (4.94)	175 (4.52)	81 (6.19)	
• Trauma	909 (17.55)	666 (17.2)	243 (18.58)	
• Congenital and tumor	138 (2.66)	103 (2.66)	35 (2.68)	
• Bullous keratopathy	1687 (32.57)	1197 (30.91)	490 (37.46)	
• Infectious Keratitis	891 (17.2)	689 (17.79)	202 (15.44)	
• Corneal opacity	540 (10.42)	408 (10.54)	132 (10.09)	
• Others	50 (0.97)	37 (0.96)	13 (0.99)	
**DM**	814 (15.71)	592 (15.29)	222 (16.97)	0.1481
**HTN**	1593 (30.75)	1147 (29.62)	446 (34.1)	0.0024
**Dyslipidemia**	950 (18.34)	699 (18.05)	251 (19.19)	0.3583
**Stroke**	20 (0.39)	15 (0.39)	5 (0.38)	0.9794
**MI**	16 (0.31)	12 (0.31)	4 (0.31)	0.9815

DM = diabetes mellitus; HTN = hypertension; MI = myocardial infarction.

*p-value was achieved by chi-square test.

**Fig 2:**
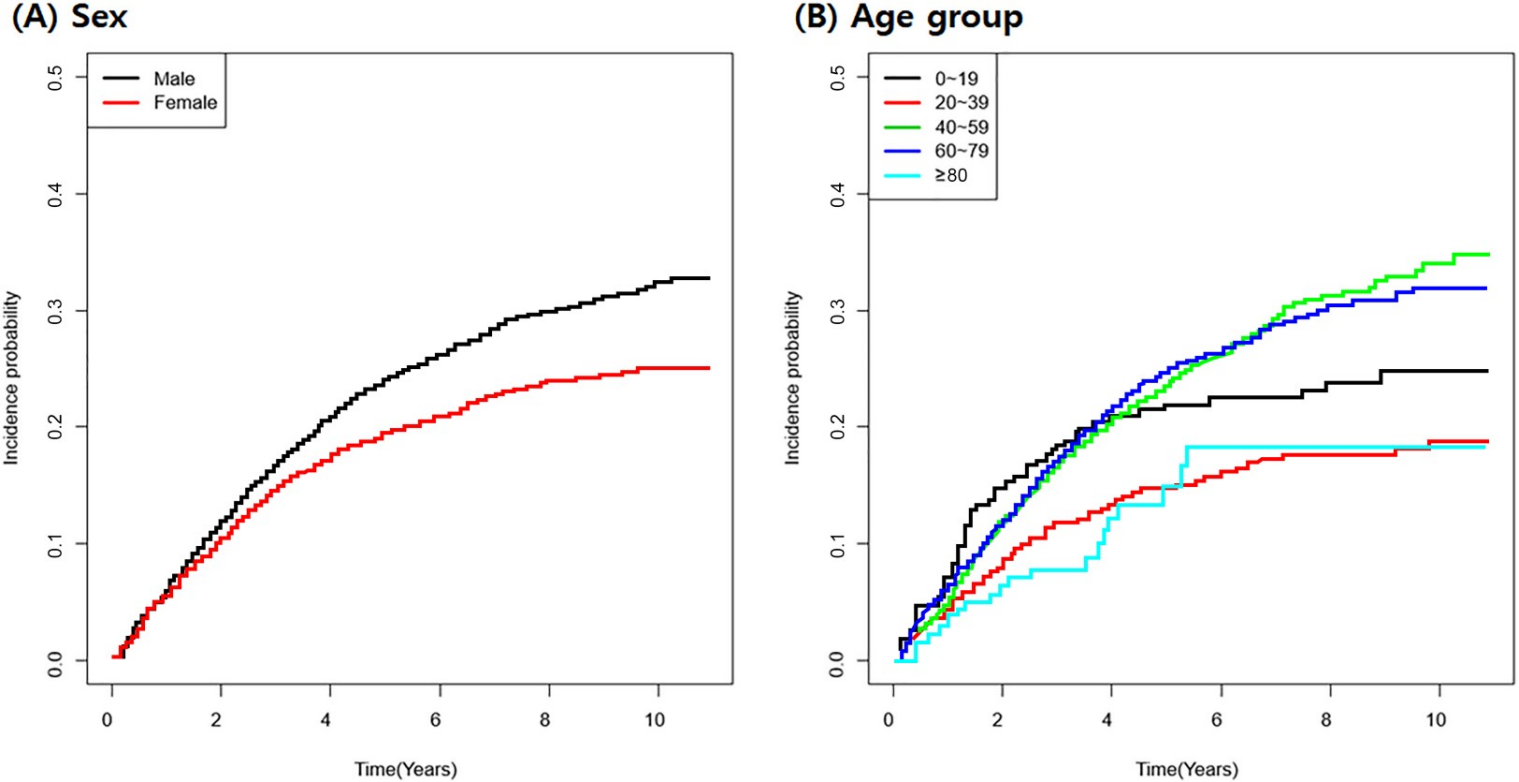
Kaplan-Meyer curve for repeated corneal transplantation by sex (A) and age group (B). Females had a lower incidence of repeated corneal transplantation, and the age groups of 40–59 and 60–79 years showed greater incidence of repeated corneal transplantation.

Table 3 demonstrates the increased risk of repeated keratoplasty by various risk factors calculated by Cox regression analysis. The average incidence of repeated keratoplasty among those who underwent corneal transplantation at least once was 43.24 per 1,000 person-year. The age group of 20 to 39 years demonstrated the lowest incidence of repeated keratoplasty (24.94 per 1,000 person-year) compared to other age groups. Females had lower incidence of repeated keratoplasty compared to males (males: 47.66 per 1,000 person-year, females: 36.04 per 1,000 person-year) with a significantly lower risk of repeated keratoplasty compared to males (HR, 0.786; CI, 0.704–0.877; *p* <0.001). Keratoconus showed significantly lower hazard ratio compared to other surgical indications of keratoplasty. Fig 2 includes Kaplan-Meyer curves for repeated keratoplasty according to sex and age group.

**Table 3:** Incidence and risk factors of repeated corneal transplantation using Cox regression analysis.

	N	Event	IR*	HR^†^
Overall	5180	1308	43.2385	
**Age**				
-19	287	68	35.5821	1.000 (ref.)
20–39	892	151	24.9407	0.690 (0.518, 0.919)
40–59	1688	489	49.1753	1.297 (1.006, 1.671)
60–79	2156	576	49.6057	1.263 (0.982, 1.625)
80-	157	24	32.8863	0.787 (0.494, 1.253)
**Sex**				
• Male	3267	893	47.6647	1.000 (ref.)
• Female	1913	415	36.0375	0.769 (0.684, 0.864)
**Income**				
• High	3913	1003	43.8725	1.000 (ref.)
• Low	1267	305	41.2768	0.958 (0.843, 1.089)
**Initial Surgical Method**				
• Lamellar keratoplasty	743	173	43.4553	1.000 (ref.)
• Penetrating keratoplasty	4437	1135	43.2056	0.998 (0.85, 1.171)
**Indication**				
• Keratoconus	709	112	22.8175	1.000 (ref.)
• Dystrophy	256	81	61.933	2.448 (1.827, 3.279)
• Trauma	909	243	48.2353	1.82 (1.444, 2.293)
• Congenital & Tumor	138	35	43.3689	1.902 (1.3, 2.781)
• Bullous Keratopathy	1687	490	53.0917	1.97 (1.577, 2.461)
• Infectious Keratitis	891	202	38.7646	1.572 (1.235, 2.003)
• Corneal opacity	540	132	38.1809	1.615 (1.247, 2.092)
• Others	50	13	44.4988	1.84 (1.032, 3.279)
**DM**				
• No	4366	1086	41.758	1.000 (ref.)
• Yes	814	222	52.3116	1.08 (0.93, 1.253)
**HTN**				
• No	3587	862	39.6239	1.000 (ref.)
• Yes	1593	446	52.4935	1.154 (1.018, 1.309)
**Dyslipidemia**				
• No	4230	1057	41.848	1.000 (ref.)
• Yes	950	251	50.2728	1.051 (0.911, 1.212)
**Stroke**				
• No	5160	1303	43.2253	1.000 (ref.)
• Yes	20	5	46.9755	1.065 (0.443, 2.56)
**MI**				
• No	5164	1304	43.2326	1.000 (ref.)
• Yes	16	4	45.2461	1.12 (0.42, 2.99)

IR = incidence rate; HR = hazard ratio; DM = diabetes mellitus; HTN = hypertension; MI = myocardial infarction.

*per 1,000 person-year.

^†^adjusted for age and sex.

## Discussion

This representative retrospective health care registry study provided an overview of the number of corneal transplants in South Korea, as well as trends in surgery type and recipient age of corneal transplantation. The study further characterized the rate of repeated corneal transplantation and risk factors for repeated surgery.

In the current study, the annual incidence of corneal transplantation ranged from 1.4488 to 1.9516 cases per 100,000 people during the study period, which is similar to the previously reported rate [2]. South Korea has a relatively lower rate of corneal transplantation compared to other developed Western countries. The United States has a 40-fold higher rate of corneal transplantation [8], and a German study found that the number of corneal transplants there was 10 times greater than that of South Korea despite the total population of German being only 1.7 times greater than that of South Korea [9]. Japan has a similar corneal transplantation rate to South Korea, possibly due to similar pools of corneal diseases and socioeconomic factors [10].

Interestingly, within corneal transplantation, the proportion of penetrating keratoplasty was relatively high in South Korea compared to other countries. In our study, the proportion of penetrating keratoplasty cases among total corneal transplantation cases was about 80–90% during the study period, although the proportion was getting decreased. There are several possible explanation for this. First, the main corneal diseases that require corneal transplantation are different in South Korea compared to other countries. Koreans have less corneal endothelial dystrophy (e.g., Fuchs’ dystrophy) compared to Western countries and less keratoconus compared to the Middle East. Corneal diseases (dystrophies and keratoconus) that can be treated with lamellar keratoplasty are relatively rare in Korea; thus, this different pool of corneal diseases might have contributed to the type of corneal transplantation. Second, the incidence of corneal transplantation is lower in Korea because the lack of donated corneal tissue. In South Korea, only Korean Netwrok for Organ Sharing (KONOS) by Korean Center for Disease Control and Prevention (KCDC) has the right to manage the donated corneal tissue, and private eyebank system to deal with corneal tissue commercially is prohibited. So only a few patients with late staged or severe status of disease can have an opportunity to get the surgery. In other words, the forms of corneal diseases are more severe, leading surgeons to perform full thickness corneal transplantation rather than lamellar keratoplasty. Third, the eyebank system in South Korea might have influenced the surgeons to perform more penetrating keratoplasty than lamellar keratoplasty. The Korean medical law does not allow technician to process the cornea. The surgeon should conduct all process of donated corneal tissue from enucleation to lamellar dissection by themselves to get lamellar tissue in order to perform lamellar keratoplasty. Keratoplasty using pre-processed imported corneal tissue can be an alternative, however, an expensive tissue costs are another obstacle. Finally, the severity of the underlying diseases, the high cost of imported tissue or disturbance on tissue processing by surgeon themselves result in higher proportion of penetrating keratoplasty rather than lamellar keratoplasty.

We found that the incidence of repeated corneal transplantation in South Korea was 43.24 per 1,000 person-years. Repeated corneal transplantation occurred in 1,308 out of 5,180 participants, with an average follow-up period of 5.47 years. Previous reports in various countries have described lower rates of repeated corneal transplantation compared to our results. Weisbrod et al. reported 116 cases of repeated penetrating keratoplasty out of 696 subjects with previous penetrating keratoplasty with a follow-up period of 7.5 years [11]. Zare et al. reported 72 eyes with repeated corneal transplantation out of 1624 eyes during a study period of 6 years [12]. Interestingly, the rate of repeated corneal transplantation in South Korea is comparable to or greater than that of other countries, while the incidence of corneal transplantation itself is much lower than that of other countries. This difference might be because keratoplasty in South Korea is performed selectively for severely progressive diseases, as stated above. As a result, there were fewer cases of keratoplasty and a greater rate of corneal graft failure and repeated keratoplasty.

Ages 20–39 years and female sex had a lower risk of repeated keratoplasty in this study. The lower risk among the age group of 20–39 years may be attributable to the etiology of keratoplasty in that age group. Keratoconus, which has good prognosis after keratoplasty [13], might make up a greater proportion of cases in the 20–30 year age group compared to other age groups, leading to lower risk of repeated corneal transplantation. The finding that females have a lower risk of repeated keratoplasty has not been reported previously in the literature. The reason for the result is not clear; females might have fewer resources or support to undergo additional surgeries compared to males or might have an etiology with more favorable outcomes than males. Unfortunately, the current study lacked sufficiently detailed clinical information on the participants to allow consideration of these points, and further studies are needed.

The strengths of this study include the large sample size and completeness of the study population. This study includes the largest population studied for corneal transplantation, and the completeness of the population is evidenced by the unique and ideal registration of the entire South Korean population under the National Health Insurance System.

However, the current study also had certain limitations. The biggest drawback is that there was no surgical code to distinguish endothelial keratoplasty from anterior lamellar keratoplasty before 2015 in South Korea. The surgical code for lamellar keratoplasty included all kinds of lamellar keratoplasty before then. From 2015, surgical code for anterior lamellar keratoplasty (S5372) and endothelial lamellar keratoplasty (S5374) were separated and assessed independently. To investigate the characteristics of endothelial keratoplasty only, much longer study period is needed from now on. A second limitation is that we were unable to access data prior to 2006, which may have decreased the accuracy of estimation of initial and repeated corneal transplantation. We regarded patients with a diagnostic code of corneal transplantation status (Z947) before the first surgery during the study period (2006 to 2016) to have received keratoplasty before 2006. However, there is a possibility that some may have undergone keratoplasty before 2006 without a diagnostic code for corneal transplantation in their records. We might have missed some patients and classified repeated cases of keratoplasty as initial cases of keratoplasty. This is an intrinsic drawback of claim data-based epidemiologic studies. Nonetheless, the annual incidence of repeated corneal transplantation in our study was relatively constant from 2006 through 2016, supporting the veracity of the incidence of repeated keratoplasty in the current study. Finally, a lack of clinical information, including detailed demographics and ophthalmologic status such as visual acuity and severity of corneal diseases, is another inherent limitation of the present study. Further studies investigating those points are warranted.

In conclusion, this is a nationwide, population-based study of the entire South Korean population highlighting the incidence of corneal transplantation. The study provides detailed trends of the incidence of corneal transplantation and rates of repeated corneal transplantation from 2006 to 2016. Men received more keratoplasty compared to women, and the proportion of lamellar keratoplasty increased from 2006 through 2016. The study also found that repeated corneal transplantation was more common in male patients, and those who received initial corneal transplantation at the ages of 20–39 years tended to have lower risk of repeated corneal transplantation. By providing a better understanding of the current status of keratoplasty, our findings can help predict socioeconomic burden and assist in planning healthcare systems accordingly.

## References

[1] Whitcher JP, Srinivasan M, Upadhyay MP. Corneal blindness: a global perspective. Bull World Health Organ 2001;79:214–21. 11285665 PMC2566379

[2] Gain P, Jullienne R, He Z, et al Global Survey of Corneal Transplantation and Eye Banking. JAMA Ophthalmol 2016;134:167–73. 10.1001/jamaophthalmol.2015.4776 26633035

[3] Eghrari AO, Gottsch JD. Fuchs' corneal dystrophy. Expert Rev Ophthalmol 2010;5:147–59. 10.1586/eop.10.8 20625449 PMC2897712

[4] Kok YO, Tan GF, Loon SC. Review: keratoconus in Asia. Cornea 2012;31:581–93. 10.1097/ICO.0b013e31820cd61d 22314815

[5] Tan DT, Janardhanan P, Zhou H, et al Penetrating keratoplasty in Asian eyes: the Singapore Corneal Transplant Study. Ophthalmology 2008;115:975–82 e1. 10.1016/j.ophtha.2007.08.049 18061267

[6] Shin DW, Cho B, Guallar E. Korean National Health Insurance Database. JAMA Intern Med 2016;176:138. 10.1001/jamainternmed.2015.7110 26747667

[7] Cheol Seong S, Kim YY, Khang YH, et al Data Resource Profile: The National Health Information Database of the National Health Insurance Service in South Korea. Int J Epidemiol 2017;46:799–800. 10.1093/ije/dyw253 27794523 PMC5837262

[8] Park CY, Lee JK, Gore PK, et al Keratoplasty in the United States: A 10-Year Review from 2005 through 2014. Ophthalmology 2015;122:2432–42. 10.1016/j.ophtha.2015.08.017 26386848

[9] Flockerzi E, Maier P, Bohringer D, et al Trends in Corneal Transplantation from 2001 to 2016 in Germany: A Report of the DOG-Section Cornea and its Keratoplasty Registry. Am J Ophthalmol 2018;188:91–8. 10.1016/j.ajo.2018.01.018 29410297

[10] Shimazaki J, Amano S, Uno T, et al National survey on bullous keratopathy in Japan. Cornea 2007;26:274–8. 10.1097/ICO.0b013e31802c9e19 17413952

[11] Weisbrod DJ, Sit M, Naor J, Slomovic AR. Outcomes of repeat penetrating keratoplasty and risk factors for graft failure. Cornea 2003;22:429–34. 10.1097/00003226-200307000-00008 12827048

[12] Zare M, Aslani M, Azimzadeh A, et al Indications and Outcomes of Repeat Penetrating Keratoplasty. Iranian Journal of Ophthalmology 2011;23:44–50.

[13] Paglen PG, Fine M, Abbott RL, Webster RG Jr., The prognosis for keratoplasty in keratoconus. Ophthalmology 1982;89:651–4. 10.1016/s0161-6420(82)34753-3 6750489

